# Associations between sheep farmer attitudes, beliefs, emotions and personality, and their barriers to uptake of best practice: The example of footrot

**DOI:** 10.1016/j.prevetmed.2016.05.009

**Published:** 2017-04-01

**Authors:** Holly O’Kane, Eamonn Ferguson, Jasmeet Kaler, Laura Green

**Affiliations:** a School of Life Sciences, University of Warwick, Gibbet Hill Road, Coventry CV4 7AL, UK; b School of Psychology, University of Nottingham, University Park, Nottingham NG7 2RD, UK; c School of Veterinary Medicine and Science, University of Nottingham, Sutton Bonington Campus, Sutton Bonington, Leicestershire LE12 5RD, UK

**Keywords:** Sheep farmer, Emotions, Attitudes, Personality, Barriers, Disease control

## Abstract

There is interest in understanding how farmers’ behaviour influences their management of livestock. We extend the theory of planned behaviour with farmers attitudes, beliefs, emotions and personality to investigate how these are associated with management of livestock disease using the example of footrot (FR) in sheep.

In May 2013 a one-year retrospective questionnaire was sent to 4000 sheep farmers in England, requesting data on lameness prevalence, management of footrot, farm/flock descriptors, and farmer-orientated themes: barriers to treating footrot, opinions and knowledge of footrot, relating to other people and personality. Principal component analysis (PCA) was used to make composite variables from explanatory variables and latent class (LC) analysis was used to subgroup farmers, based on nine managements of FR. Associations between LC and composite variables were investigated using multinomial logistic regression. Negative binomial regression was used to investigate associations between the proportion of lame sheep and composite and personality variables.

The useable response rate was 32% and 97% of farmers reported having lame sheep; the geometric mean prevalence of lameness (GMPL) was 3.7% (95% CI 3.51%–3.86%).

Participants grouped into three latent classes; LC1 (best practice—treat FR within 3 days of sheep becoming lame; use injectable and topical antibiotics; avoid foot trimming), 11% farmers), LC2 (slow to act, 57%) and LC3 (slow to act, delayed culling, 32%), with GMPL 2.95%, 3.60% and 4.10% respectively.

Farmers who reported the production cycle as a barrier to treating sheep with FR were more likely to be in LC2 (RRR 1.36) than LC1. Negative emotions towards FR were associated with higher risk of being in LC2 (RRR 1.39) than LC1. Knowledge of preventing FR spread was associated with a lower risk of being in LC2 (RRR 0.46) or LC3 (RRR 0.34) than LC1. Knowledge about FR transmission was associated with a lower risk of being in LC3 (RRR 0.64) than LC1.

An increased risk of lameness was associated with the production cycle being a barrier to treating sheep with FR (IRR 1.13), negative emotions towards FR (IRR 1.13) and feelings of hopelessness towards FR (IRR 1.20). Conscientiousness (IRR 0.95) and understanding the importance of active control of lameness (IRR 0.76) were associated with reduced risk of lameness.

We conclude that emotions and personality are associated with differences in farmer management of FR and prevalence of lameness. Further understanding how personality and emotions influence change in behaviour is key to increasing uptake of new information.

## 1 Introduction

There is increasing interest in understanding farmer behaviour with respect to management of their livestock. Whilst farmers are likely to be interested in making profits (Gasson et al., 1993) and increasing production (Ploeg, 1993), farmers are not simply rational profit maximisers and are likely to have emotional reactions to their animals that also influence decision making (Bigras-Poulin et al., 1985). Thus, rural sociologists have applied theories like the theory of reasoned action (TRA) (Fishbein and Ajzen, 1975) and its extension, the theory of planned behaviour (TPB) (Ajzen, 1991) to understand farmers’ decisions and explain variability in the health and welfare of the animals in their care (Hemsworth et al., 1989, Willock et al., 1999, Garforth et al., 2006, Jansen et al., 2009, Kauppinen et al., 2012). Whilst useful, such models still focus on rational processes around attitudes and beliefs. In this paper we extend these to look at a wider spectrum of emotional factors such as the type and extent of specific emotional responses such as sadness or anger expressed by farmers’ to their animals’ disease, the Big-5 personality factors (Goldberg, 1993) and trait empathy (Davis, 1983). These have all been shown to have strong effects on human decision-making (Damasio et al., 1996, Bechara et al., 1997, Loewenstein et al., 2001, Dolan, 2002, De Martino et al., 2006, Ferguson et al., 2009) and influence decisions across a wide range of human activity; relationships, health, employment, medical decision making etc. but to date have not been explored in a livestock context (Hunter and Hunter, 1984, Ferguson et al., 2003, Roberts et al., 2007, Ferguson, 2013, Molloy et al., 2014).

According to the TPB, the best predictor of behaviour is a person’s intention to act, and empirical evidence supports this (Webb and Sheeran, 2006). Intention to act is predicted by attitudes towards a behaviour (whether the behaviour is viewed as desirable or not), subjective norms (whether significant others think it is worth pursuing or not), perceived behavioural control (the ability to perform the behaviour), perception of resources and opportunities to perform, or obstacles to avoid, a behaviour. Using TPB alone to predict behaviour, however, oversimplifies the complexities of decision-making. For example, the key role of emotional processing and personality are overlooked. Here we examine traits (feelings in general) of empathy and normal human personality as well as more process oriented indices of emotions that focus on how the farmer feels about their flock.

There is now a substantive body of evidence to support the central and crucial role of such emotional experiences and processes (both positive and negative) in human decision making (Damasio et al., 1996, Bechara et al., 1997, Loewenstein et al., 2001, Dolan, 2002, De Martino et al., 2006, Ferguson et al., 2009). Theoretical models show that traits act to influence how such emotional processes operate to influence final decisions (Ferguson et al., 2011, Ferguson, 2013). Thus a person who is high in empathy is more likely to feel compassion towards an individual and act to help them (Batson, 2014, Ferguson, 2015). Empathy has been studied in relation to farmer behaviour previously (Kielland et al., 2010). These authors reported that farmers who perceive that animals feel pain as humans do have greater empathy towards their cattle and better welfare outcomes on their farms. Here we extend this to general traits of empathy. Trait empathy can be split fundamentally into cognitive empathy that includes perspective taking (imagining how the other is feeling) and affective empathy (feeling for the target) (Davis, 1983, Ferguson, 2015) and we explore whether these two traits differentially predict animal welfare using the example of footrot in sheep.

The dominant conceptualization of human personality is based on five broad domains in terms of degrees of extraversion (high scores equate to outgoing and sensation seeking), agreeableness (high scores equate altruism and caring), conscientiousness (high scores equate to hardworking and being methodical), emotional stability (high scores equate to being calm) and openness to experiences (high scores equate to being artistic and seeking out new cultures) (Gosling et al., 2003). In the literature, conscientiousness is consistently the strongest positive predictor of performance across a wide number of domains (Salgado, 1997, Bogg and Roberts, 2004), and we hypothesise that it should be the case here. Agreeableness may influence emotions of compassion and caring towards an animal and thus may also predict positive animal health. We examine these within the context of footrot. Identifying traits associated with good practice has implications for interventions because modern personality theory (Roberts and Jackson, 2008), supported by a substantial body of evidence shows that personality traits can change both over time and in response to environmental challenges and specific training (Caspi et al., 2005, Roberts et al., 2013, Hudson and Fraley, 2015).

Lameness is a major welfare concern for both sheep farmers and veterinarians (Fitzpatrick et al., 2006, Goddard et al., 2006; Morgan-Davies et al., 2006). In 2004 the global period prevalence of lameness in England was 10.6% (Kaler and Green, 2008), with more than 90% of lameness attributed to footrot. Footrot is an infectious disease caused by *Dichelobacter nodosus* (Witcomb et al., 2015). It has two clinical presentations, inflammation of the interdigital skin of the foot (interdigital dermatitis) and separation of the hoof horn from the underlying tissue (severe footrot). From the 1950s–1990s prevention of footrot focused on whole flock managements such as quarantine of new and diseased sheep, routine foot trimming and foot bathing and vaccination (e.g. Morgan, 1987). More recently, research has indicated that prompt treatment of individual sheep lame with footrot with parenteral and topical antibiotics without foot trimming reduces the duration of disease (Kaler et al., 2010a, Kaler et al., 2010b, Wassink et al., 2010), reduces recurrence (Kaler et al., 2010b), protects flock mates (Green et al., 2007) and so reduces the incidence and prevalence of lameness. Whole flock managements of quarantine (Wassink et al., 2004, Winter et al., 2015) and vaccination (Winter et al., 2015) are also associated with a lower prevalence of footrot. In contrast, routine foot trimming and foot bathing are associated with a higher prevalence of footrot (Wassink et al., 2005, Green et al., 2007, Kaler and Green, 2008, King, 2013, Winter et al., 2015).

In 2013 a questionnaire was sent to 4000 English sheep farmers. The questionnaire focused on management of footrot and attitudes and emotions towards lameness and footrot and farmer personality traits. From analysis of 1260 respondents, management factors associated with a lower prevalence of lameness included quarantine of incoming sheep for >3 weeks, recognising very mild lameness in sheep (locomotion score 1 Kaler et al., 2009), treating lame sheep within 3 days, treating the first lame sheep in a group compared with treating when >5 sheep were lame, vaccination against footrot and selecting replacements from never lame ewes. Factors associated with a higher prevalence of lameness were feet bleeding at routine foot trimming and difficulty catching individual lame sheep. Factors associated with lower prevalence of lameness in the sub model on treatment of footrot were using parenteral and topical antibacterials and avoiding foot trimming. From the sub-model on culling, waiting until ewes were persistently lame before culling was associated with higher prevalence of lameness (Winter et al., 2015).

In this paper we analyse further data on attitudes, beliefs, emotions and personality together with management of footrot from the farmers who responded to the 2013 questionnaire (Winter et al., 2015). The objectives of this study were to test whether we can identify sub-groups of farmers who apply footrot management activities differently and to test the hypothesis that farmer personality, emotions, empathy and attitudes and beliefs about footrot are associated with different approaches to management of footrot and consequently to the prevalence of lameness.

## 2 Materials and methods

### 2.1 Study sample

4000 English lowland flocks, from a total of 15000, targeted to have at least 200 ewes were randomly selected by Defra (Department for Environment, Food and Rural Affairs) from the 2010 agricultural census and from the AHDB Beef & Lamb Better Returns Programme.

### 2.2 Questionnaire design

A 16-page self-administered postal questionnaire was designed to capture data for the period May 2012 to April 2013 on farmer recognition of lameness and foot lesions, period prevalence of lameness, management of footrot (including both interdigital dermatitis (ID) and severe footrot (SFR)) and farm and flock descriptors. The responses to this section of the questionnaire have been analysed and reported (Winter et al., 2015) and summarised in the introduction. A second section was designed to capture data on 5 themes based on models of emotion and traits detailed in the introduction: Theme 1 (barriers to treating footrot) contained ten statements reflecting ideas in the TPB around barriers to treatment. Statements in theme 1 focused specifically on scenarios which may prevent farmers from treating sheep with footrot, e.g. “Lack of time prevents me from treating a sheep as soon as I see it lame”. Theme 2 (beliefs about footrot) focused on farmers’ beliefs and emotions towards footrot. It contained nineteen statements adapted from the Illness Perceptions Questionnaire-Revised (IPQ-R) for human health (Moss-Morris et al., 2002) to investigate illness perceptions and emotional reactions of farmers to footrot in their sheep, for example “the number of sheep with this disease in my flock affects how many lambs I produce” and “having this disease in my flock makes me feel angry.” Theme 3 (knowledge about the aetiology of footrot) contained twenty-one statements adapted from the IPQ-R (Moss-Morris et al., 2002) to investigate farmers’ perceived causal model of footrot, for example “this disease is caused by bacteria”. Theme 4 (relating to others) contained thirteen statements to measure trait empathy using scales of the interpersonal reaction inventory (IRI) (Davis, 1983). These statements included “I often have tender, concerned feelings for people less fortunate than me” and “other people’s misfortunes do not usually disturb me a great deal” to measure empathic concern and perspective taking. Theme 5 (about you) contained ten paired personality characteristics to measure the Big-5, using the ten-item-personality-inventory (TIPI) (Gosling et al., 2003). For themed sections 1–4 farmers were asked to respond to statements using a 5–point Likert-type scale where 1 = strongly disagree and 5 = strongly agree. For theme 5, farmers were asked to respond to statements using a 7–point Likert-type scale where 1 = strongly disagree and 7 = strongly agree; where an “other” response was considered appropriate, space was allowed for free text.

The questionnaire was piloted on 20 sheep farmers; three responded. Respondents reported taking 30, 40 and 45 min to complete the questionnaire and all comments about the questionnaire were positive.

### 2.3 Recruitment of participants

Recruitment is detailed in Winter et al. (2015), briefly, a letter introducing the study was sent to 4000 farmers in May 2013 informing them that they had been selected for the study and that a questionnaire would be sent to them within 10–14 days. The questionnaire was sent in June together with a cover letter, study information and a prepaid return envelope. Farmers were invited to participate in a free draw with 5 winners each receiving a £50 shopping voucher. Reminder postcards were sent to non-respondents on the 18th June 2013 and 8th July 2013, a new questionnaire was sent to non-respondents with the second reminder. Thank you postcards were sent to respondents. Each questionnaire was allocated a unique ID number which was printed on the first and last page of the questionnaire. Double data entry was undertaken by an external agency (Wyman Dillon Ltd.). Data were stored in Microsoft Excel and cleaned using specifically written code (Winter et al., 2015).

### 2.4 Data analysis

Farmers were excluded from all analyses if details of flock size or average prevalence of lameness in ewes was not provided. Data were analysed in StataSE13 (StataCorp. 2013), SPSS 22 and MPlus 7 (Muthén and Muthén, 1998–2012). The geometric mean (GM) year period prevalence of lameness and median flock size were estimated. Frequency distributions of responses to each statement in the five themes were obtained.

The Likert scores were treated as continuous variables. Items were reverse scored where necessary (Table 1) so that for all variables high scores equated to increased quantity of the measure (e.g. greater sadness).

#### 2.4.1 Measuring farmer personality

The TIPI responses were scored using methods outlined by Gosling et al. (2003) to obtain measures of the Big-Five personality domains: extraversion, agreeableness, conscientiousness, emotional stability and openness to experiences.

#### 2.4.2 Measuring farmer empathy

Responses to the 13 statements from two of the IRI sub-scales “Empathic Concern” (7 items assessing the extent to which the farmer generally expresses sympathy/compassion for others distress) and “Perspective Taking” (6 items assessing the extent to which the farmer generally takes into account the perspective of others) were scored and reverse scored as specified by Davis (1980). One item was missing from the “Perspective Taking” due to an administrative error. Individual farmer measures for each subscale were then obtained by taking the cumulative score for those items on each subscale.

#### 2.4.3 Latent class analysis of farmer behaviour of management of lameness and footrot in their flock

We identified 15 categories within nine managements for footrot associated with the incidence or prevalence of footrot from recent research (Wassink et al., 2005, Wassink et al., 2010, Green et al., 2007, Kaler and Green, 2009, Kaler et al., 2010a, Kaler et al., 2010b, King, 2013, Winter et al., 2015). The nine variables were: (1) treat sheep within three days of seeing them lame (1 = *yes*, 0 = *no*), (2) number of sheep lame in a group before treatment (1 = 1, 2 = 2–5, 3 = 6+), (3) trim the feet of lambs (highly correlated to ewes) with footrot (0 = *yes*, 1 = *no*), (4) ability to correctly name ID and footrot (0 = *no*, 1 = *yes*), (5) always treat footrot with parenteral antibiotic (0 = *no*, 1 = *yes*), (6) and foot spray (0 = *no*, 1 = *yes*), (7) identify sheep for culling because of lameness by memory, (0 = *yes*, 1 = *no*) (8) number of episodes of lameness before culling (1 = *did not cull*, 2 = *once*, 3 *=* *twice,* 4 = > *twice*) and (9) vaccinate ewes against footrot *(0* *=* *no, 1* *=* *yes)*. We used latent class analysis (LCA) in M*Plus* 7 (Muthén and Muthén, 1998–2012) to determine the number of subgroups of farmers on the basis of these 15 variables. Latent class models ranging from two-classes to four-classes were first obtained (Muthén and Muthén, 1998–2012). To increase confidence that the final solution for each model had converged on the global maximum solution, models were repeatedly estimated with increasing random start values until the log likelihood was replicated several times (Nylund et al., 2007). Goodness-of-fit statistics (AIC, BIC, Entropy, LMR, BLRT) as outlined by (Weich et al., 2011) combined with intuitive reasoning were used to select the final latent class model. Upon reaching a final solution, the posterior probability for each farmer being in each class and then the conditional probability that farmers in a class were practising a management were calculated.

The geometric mean period prevalence of lameness and 95% CI were calculated for each latent class. The association between period prevalence of lameness and latent class membership was assessed using the Kruskal Wallis test.

#### 2.4.4 Principal component analysis of farmer beliefs, attitudes and emotions towards footrot, sheep and people

**Fig. 1:**
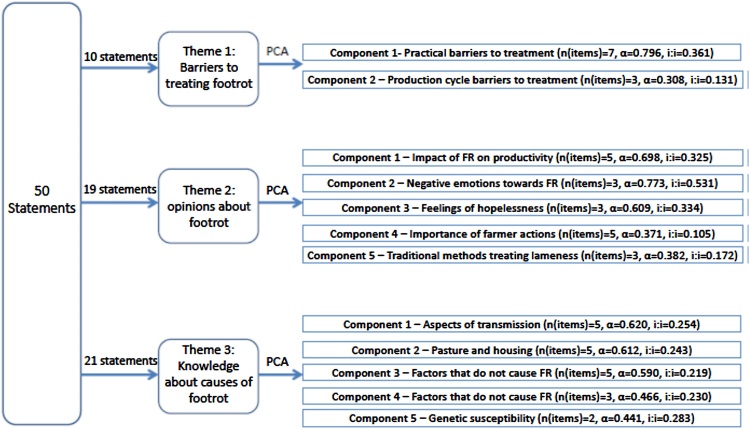
Flow diagram of the application of principal component analysis (PCA) of 1294 English sheep farmer responses to questionnaire statements by theme, the components derived from each PCA and measures of scale reliability for each component (Cronbach’s alpha (α) and mean inter-item correlation (i:i)).

Separate exploratory principal component analyses (PCA) were conducted on statements within themes 1–3 of the questionnaire (Fig. 1) using SPSS version 22. For groups of themed statements, a simple final solution was selected using a combination of the K1 rule, Scree plot, parallel analysis and theoretical and conceptual coherence (Ferguson and Cox, 1993). Parallel analysis was conducted using adapted prewritten syntax (O’Connor, 2000). The Scree and parallel analysis plots were used as the main decision aids because they are the most reliable and accurate, however, when these tools did not present logical components, the solution presenting the best conceptual coherence was selected. Statements with loadings ≥0.3 on their target factor were retained. Statements which did not load ≥0.3 on any component were removed and the PCA was repeated. Cronbach’s coefficients alpha (α) and mean inter-item correlations (i:i) were calculated for each component to assess the internal reliability of each scale, with α = 0.6-0.8 and i:i = 0.2–0.4 representing a scale with sufficient internal consistency (Ferguson and Cox, 1993). Given that α is influenced by the number of items included in a component (as n increases, α increases), a low α may occur due to a small number of items on a scale in which case an i:i of 0.2–0.4 is appropriate. Where neither of these measures met the criteria for internal consistency for a component and it was therefore theoretically unreliable, the components were assessed for conceptual coherence.

The unit scores for each component obtained by the PCA were calculated by taking the arithmetic mean of the item scores for that component. Farmers who did not respond to all items for a given component were classed as having a missing value for that component.

#### 2.4.5 Modelling the relationship between management of footrot or prevalence of lameness and farmer attitudes, beliefs, emotions and personality

Two models were developed:

Multinomial logistic regression modelling was conducted in STATA SE13. The model took the form:

Logit(π_1k_/p_i0k_) = β_0k_ + ∑ β_0_x + *e*_K_

(1) Logit(π_2k_/p_i0k_) = β_1k_ + ∑ β_1_x + *e*_K_

where logit(π_1_/p_i0_) = the probability of latent class (LC)2 versus LC1 and logit(π_2_/p_i0_) = the probability of LC3 versus LC1, β0_k_ and β_1k_ are constants for LC2 and LC3, β_0_x and β_1_x are the series of coefficients of exploratory variables X for LC2 and LC2, and e_k_ is the residual variance fixed to a binomial distribution.

Separate univariable models were built for each exploratory variable derived from the PCAs and TIPI; variables with a P value ≤0.2 (Supplementary Table 1) were tested in a multivariable model built using manual forward stepwise selection (Dohoo et al., 2003). After an initial model was built, all variables regardless of their significance at the univariable level, were tested to check for residual confounding (Cox and Wermuth, 1996). Variables with p ≤ 0.05 for at least one latent class compared with the baseline group (LC1) were left in the final model.

A negative binomial regression model was developed in STATA SE 13. The model took the form:

(2) Number of lame ewes on farm ∼ α + offset + βiXi + e

(∼ = log link function, α is the intercept, offset is the natural log of the flock size, βi is the coefficients for a series of exploratory variables, Xi, and e is the residual error).

Again, separate univariable models were built for each exploratory variable derived from the PCAs and TIPI; variables (Supplementary Table 2) with a P value ≤0.2 were identified for inclusion within a multivariable model. A multivariable model was built using forward stepwise selection (Dohoo et al., 2003). All remaining variables were retested in the multivariable model to check for residual confounding. Explanatory variables with *p*-values ≤0.05 using Wald’s statistic, were left in the final model. The model fits were explored.

## 3 Results

The useable response proportion was 1294 (32%), although not all farmers answered all questions. The number of ewes per flock ranged from 2 to 6000 (median 340) and 97% of farmers reported having lame sheep. The geometric mean year period prevalence of lameness in ewes was 3.7% (95% CI 3.51%–3.86%).

### 3.1 Measuring farmer personality

Frequency distributions for the final TIPI scores are presented in Supplementary Fig. 1. TIPI Scores for all five personality traits were reasonably normally distributed with a high kurtosis at score 4 (neither agree nor disagree) and with a slight left skew.

### 3.2 Farmers empathy scores

“Empathic concern” and “perspective taking” cumulative scores had a mean (standard deviation) of 18.14 (4.3) and 14.2 (3.7) with a range of 3–29 (possible 1–35) and 1–25 (possible 1–30) respectively. The skewness was negative for both the sub- scales −0.05 (“Empathic concern”) and −2.5 (“Perspective taking”). Both the subscales had good internal consistency and reliability (Cronbach’s α=; 0.66 (“Empathic concern”); 0.58 (“Perspective taking”) and mean inter-item correlations = 0.22 (“Empathic concern”); 0.20 (“Perspective taking”)).

### 3.3 Latent class analysis of farmer behaviours for treatment of footrot

**Fig. 2:**
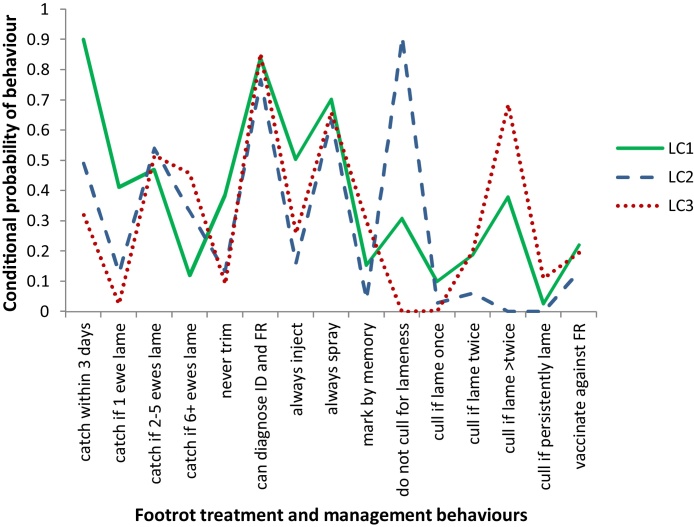
Conditional probabilities* for the occurrence of behaviours to treat and manage footrot by 1294 English sheep farmers in a three-class model. *Probability that a farmer allocated to the latent class will perform that behaviour.

The AIC decreased from the two-class model to the four-class model whereas the BIC increased as the number of classes increased. Entropy was highest for the two-class model, however, both the LMR and BLRT suggested that the three-class model was better than the two-class model (Supplementary Table 3). Taking the goodness-of-fit statistics into account and assessing each model for interpretability, the three-class model was selected (see Fig. 2). Respondents in LC1 were typical of “best practice” with the conditional probability highest in the categories indicative of best practice, with the exception of actively culling sheep that had been lame previously. Farmers in LC2 were “slow to act” with a smaller conditional probability of doing the best practice options compared with the “best practice” class. Farmers in LC3 were “slow to act, delayed culling”. This class was characterised by lowest conditional probabilities for best practice behaviours compared with the best practice and slow to act classes, with a larger conditional probability of delaying culling of persistently lame ewes (Table 1). There were 11%, 32% and 57% of respondents in the best practice, slow to act and slow to act, delayed culling classes respectively with geometric mean prevalence of lameness of 2.95%, 3.60% and 4.10% respectively. The conditional probability of each management being practiced by latent class is presented in Fig. 2 (Supplementary Table 4).

### 3.4 Principal component analysis of farmer beliefs, attitudes and emotions towards footrot, sheep and people

The 50 statements within the 1–3 themed sections of the questionnaire were reduced to fourteen components (Table 2). Theme 1 (barriers to treating footrot) contained ten statements which were reduced to two components: component (1) practical barriers to treating footrot such as difficulty catching mildly lame sheep, and (2) production cycle barriers to treating footrot including not treating ewes during tupping or which are heavily pregnant.

The nineteen statements from theme 2 were reduced to five components: (1) opinions about the impact of footrot on productivity, for example, by affecting the number of lambs born or the body condition of ewes; (2) negative emotions towards footrot such as anger and misery; (3) feelings of hopelessness towards footrot such as believing they will always have lame sheep in their flock; (4) opinions about the importance of farmer actions in tackling footrot for example treating sheep within three days of becoming lame; and (5) opinions about traditional methods of treating lameness such as foot trimming. While components 4 and 5 showed lower reliability they were conceptually coherent.

Twenty of the twenty-one statements from theme 3 were reduced to five components: (1) aspects of disease transmission for example believing footrot is caused by bacteria or keeping persistently lame sheep; (2) the role of housing and pasture in footrot, including stocking density; (3) factors which do not cause footrot such as foot bathing; (4) other factors which do not cause footrot such as body condition, and (5) genetic susceptibility to footrot such as breed or heredity.

### 3.5 Multinomial multivariable analyses of latent class membership and period prevalence of lameness and beliefs, attitudes, emotions and personality

The univariable multinomial logistic regression results are presented in Supplementary Table 1. In the final multivariable model (Table 3) compared with farmers doing best practice, farmers were more likely to be slow to act if they reported that the production cycle was a barrier to treating footrot (RRR 1.36). Farmers were more likely to be slow to act, with delayed culling (RRR 1.39) than doing best practice if they had negative emotions, that is, footrot in their flock made them feel sad or angry. Farmers were more likely to be slow to act with delayed culling (RRR 2.17) or slow to act (RRR 2.94) compared with doing best practice if they used traditional methods to treat lameness, stating that foot trimming was effective and antibiotics were ineffective. Knowledge about transmission of footrot (e.g. footrot is caused by other sheep with footrot in the flock) reduced the risk of being slow to act (RRR 0.64) compared with best practice.

The univariable negative binomial regression results are presented in Supplementary Table 2. In the multivariable model (Table 4), farmers had a higher risk of lameness in their flock if they agreed there were “production cycle barriers to treating footrot” (IRR 1.13), had “negative emotions towards footrot” (IRR 1.13) or had “feelings of hopelessness towards footrot” (IRR 1.20) than farmers without these beliefs. Farmers who believed in the “importance of early actions” had a reduced risk of lameness (0.76) as did farmers who considered themselves conscientious (IRR 0.95) than farmers without these beliefs.

## 4 Discussion

This is the first study to investigate farmer personality, emotions, empathy, attitudes and beliefs towards a livestock disease. Importantly, a mixture of non-rational cognitive processes including emotions and personality, as well as physical barriers, and knowledge about footrot, were linked to farmer management of footrot and consequently to the prevalence of lameness.

A second innovation in this paper is the use of LCA to identify sub-groups of farmers with different behavioural profiles with respect to the management of lame sheep. Our latent class analysis identified three classes of farmers based on their behavioural approaches to the treatment and control of lameness and footrot in sheep. We show that there is a best practice, compliant group that makes up 11% of the sample, with the remaining 89% made up of two non-compliant groups that differ with respect to time to treatment, type of treatment and culling strategies. While it may have been possible a-priori to identify a ‘best practice’ group, different non-compliant groups could not have been identified without latent class analysis. The heterogeneity within non-compliance is an important observation. In general, non-compliance is treated as one behaviour in a single group of people and predictors of (Molloy et al., 2014, Haskard Zolnierek and DiMatteo, 2009 and intervention for (Demonceau et al., 2013) non-compliance, compared to compliance, explored. Our findings of heterogeneity in non-compliance resonate with recent work on non-compliance in breast cancer patients (see Tinari et al., 2015). The important implication of the heterogeneity in non-compliance is that one size fits all interventions for non-compliance are most unlikely to be effective to all non-compliant farmers and interventions tailored to the problems and needs of the different non-compliant groups are needed. The stratified medicine approach, which is gaining momentum in human medicine with respect to treatment allocation, strongly suggests that efficiency and success of interventions is predicated on targeting interventions to the most appropriate groups.

The LCA results provide further support for research indicating that prompt treatment (within three days) of individual lame sheep using parenteral and topical antibiotic, not trimming the hoof (Green et al., 2007, Kaler et al., 2010a, Kaler et al., 2010b, Wassink et al., 2010) as well as vaccination, although rarely done by any LC (Winter et al., 2015) are effective in the treatment and control of lameness and footrot in sheep. The higher prevalence of lameness in flocks belonging to farmers in the less compliant groups indicates the role of prompt treatment, which is where these groups varied most from the best practice group, whilst the slow to treat delayed culling class also highlight that culling persistently lame sheep is too late, these sheep are raising the prevalence of lameness by being lame themselves and probably causing lameness in other sheep (Green et al., 2007). Late culling is not an effective strategy.

As well as associations with the prevalence of lameness, latent class membership was associated with beliefs, knowledge and emotions which can be used to gain insight into farmer cognitions, emotions and behaviours towards adopting new practices for the treatment and management of footrot. In terms of planned behaviour, barriers that led some farmers to avoid treating lame sheep at specific times of the production cycle (pregnancy and tupping for adults and finishing (fattening) period for lambs) were also less likely to use best practice and so less likely to treat sheep promptly at other times of the year. This suggests that these farmers are not prioritising lameness at any time of year and so the barrier to change might be greater than initially anticipated.

Those farmers who expressed negative emotions (feelings of frustration, anger, misery) towards footrot were more likely to be in the slow to act, delayed culling class (Table 4) and these emotions were associated with greater risk of lameness (Table 3). This further validates the distinction between the two classes of non-compliance, and shows how different interventions, based on emotions, may be important to redress non-compliance in one sub-group but not the other. It is also of interest that it is emotions that are a mix of sadness and anger that distinguish the groups. Depression has been linked to medical non-compliance (DiMatteo et al., 2000) and depression in farmers (although not specifically measured) might be a factor for non-compliance in managing footrot in the current study.

Negative emotional reactions were also linked to a higher prevalence of lameness. However, in this case it is not just sadness/anger but a sense of hopelessness. Such negative feelings are likely to result in inaction and resignation (Lazarus, 1991). Problems with lameness prevalence could escalate with farmers entering a cycle of self-fulfilling behaviour and belief with farmers practising behaviours associated with higher prevalence of lameness being more likely to have negative feelings leading to inaction and so on. In contrast, farmers who understood the importance of their own actions had a lower prevalence of lameness. This further suggests that farmers’ ability to act appropriately towards footrot is in part predicted by their perceived behavioural control and an acceptance of the consequences of their actions. Motivation as a result of perception of control has been linked to ‘good’ behaviour in previous studies of disease in livestock (Gunn et al., 2008, Jansen et al., 2009).

Surprisingly, there was no significant association between trait empathy and farmer latent class or prevalence of lameness, this may reflect that human empathy and human animal empathy might not be measured by one single or similar construct (Paul, 2000). There is a possibility that because of social desirability of the measures we were not able to capture trait empathy accurately, or there was not enough variability, for both subscales the peak of score distributions tended to be to the right side of the distribution (higher scores) i.e. farmers rated themselves positively for empathy.

Farmers who understood “aspects of transmission” and the negative impact of “traditional methods of treating lameness”, that promote maintenance and spread footrot, were more likely to be in the best practice class compared with the less compliant classes. Thus this correct knowledge that is part of the components of the farmer’s illness model for footrot, has important implications for farmer compliance with managing this disease in their own livestock.

As predicted, of the five personality domains, conscientiousness was the strongest personality predictor of performance and was associated with lower prevalence of lameness (Table 4). Of all the domains of the Big-5 model of personality (Costa and McCrae, 1992, John and Srivastava, 1999), conscientiousness has consistently been reported as the most predictive of positive behavioural outcomes and self-protective behaviours (Ferguson, 2013, Molloy et al., 2014). The organisation and order that come with high conscientiousness probably drive future planning and preparation for managing and treating footrot and conscientious farmers take time to review new evidence.

The findings from the current paper have further implications for farmer interventions.

For example, one implication here is that the ‘compliant–best practice’ class probably need to be excluded from an analysis of interventions, or randomization to treatments, because this group is unlikely to show any real significant changes in behaviour. This all implies that ‘one-size fits all’ interventions are unlikely to be effective or needed.

Also, what types of interventions are likely to be effective? These range from behaviour non-specific to behaviour specific (although these are not mutually exclusive). First, behaviour non-specific interventions that increase levels of conscientiousness could be used. There is now a substantial body of evidence to show that traits such as conscientiousness change as a function of training or environmental experience (Ludtke et al., 2011, Roberts et al., 2013). Indeed, simple motivational training procedures have been applied with some degree of success (Hudson and Fraley, 2015). So far these are all small scale and laboratory based, but it does raise the possibility of targeting conscientious behaviours to motivate change. Here the idea is that by changing some levels of conscientiousness you see wide scale benefits across many behaviours (Ferguson, 2013). Second, and moving towards a more behaviour specific type of intervention, we could target emotions that are associated with the target behaviour. Given the emotional factors linked to non-compliance, adapted cognitive behavioural therapy and guided self-help approaches for non-clinical contexts to treat emotional problems can be effective (Gellatly et al., 2007, Pleva and Wade, 2006) as can mindfulness (Cavanagh et al., 2013). The key barrier here is acceptability of such interventions for farmers. However, the strong indication from our results of the key role of emotional factors warrants further investigation.

Finally, there is misunderstanding about footrot itself. While educational materials, on their own, have not been effective in changing behaviour among some farmers, embedding these within framed messages may well be effective, in particular for the slow to act farmers who perceive barriers but who do not have the negative emotional pull (Kahneman and Tversky, 1979).

There are some considerations with the hypotheses generated above, first, farmers in this study may be different from the target population of English farmers because they were willing to participate in this study. Second, this study relied on self-reported information which is subject to many types of bias including recall bias and social desirability. Given that farmers generally perform the same practices year on year (Kaler and Green, 2009), recall bias is considered to be minimal and where farmers may have recently changed their management behaviours, such behaviours should be relatively fresh in their minds and again result in minimal recall issues. We are confident in the self-reported estimates of prevalence; sheep farmers have previously been found to estimate lameness fairly accurately with modest under estimation once prevalence exceeded 9% (King and Green, 2011). Third, some of the components retained in the multinomial and negative binomial regression models did not meet our pre-set criteria (α and i:i) for internal consistency. Given that in most cases this was likely to be a result of the limited numbers of items in those components and that the items on each component demonstrated cognitive coherence, we consider these components were suitable measures of the farmers’ attitudes and emotions and preferable to using each item in isolation. Fourth, this study describes the relationship between farmer behaviour (latent class) and predictors of behaviour (attitudes and personality) concurrently it does not determine a causal effect. Finally, lameness was associated with both negative emotions and feelings of hopelessness; the direction of this relationship is unclear from our study and a next stage would be to test whether education on the best managements and understanding of prevention of spread of disease and knowledge of risks for transmission can lead to improved uptake of best practice or whether overcoming negative beliefs and instilling a feeling of perceived control may be key in influencing a change in behaviours related to the management of footrot. Further research is needed in this area.

## Conflict of interests

There are no conflicts of interest

## Figures and Tables

**Table 1:** Number (percentage) of questionnaire responses for approximately 1294 English sheep farmers and measures of scale reliability (Cronbach’s α and mean inter-item correlations) for each component.

	Number (%) farmers selecting the option				
	1	2	3	4	5
	Strongly Disagree	Disagree	Neither agree/disagree	Agree	Strongly agree
Theme 1—Barriers to treating footrot					
Component 1: Practical barriers to treatment (α = 0.796, i:i = 0.361)					
“I have difficultly identifying and finding a mildly lame sheep once the flock is gathered.”	163 (12.8)	237 (18.6)	200 (15.7)	498 (39.1)	176 (13.8)
“The distance of the flock from suitable handling facilities prevents me from treating a sheep as soon as I see it lame.”	257 (20.0)	362 (28.2)	249 (19.4)	332 (25.9)	83 (6.5)
“Lack of an assistant to help gather sheep prevents me from treating a sheep as soon as I see it lame.”	294 (23.0)	410 (32.0)	224 (17.5)	278 (21.7)	75 (5.9)
“Lack of time prevents me from treating a sheep as soon as I see it lame.”	206 (16.1)	417 (32.7)	315 (24.7)	280 (21.9)	59 (4.6)
“I have difficulty catching mildly lame sheep in the field for treatment.”	112 (8.8)	237 (18.5)	225 (17.6)	471 (36.8)	235 (18.4)
“Lack of a suitably trained dog to gather sheep prevents me from treating a sheep as soon as I see it lame.”	331 (26.0)	451 (35.5)	2085 (16.4)	178 (14.0)	103 (8.1)
“At certain times of year I am too busy with other activities to treat lame sheep.”	211 (16.5)	420 (32.8)	297 (23.2)	288 (22.5)	63 (4.9)
Component 2: Production cycle barriers to treatment (α = 0.308 i:i = 0.131)					
“I don’t treat lame ewes when heavily pregnant.”	241 (19.0)	476 (37.4)	193 (15.2)	251 (19.7)	111 (8.7)
“I don’t use antibiotic injection to treat footrot in lambs that I am finishing for slaughter.”	122 (9.7)	297 (23.6)	242 (19.2)	341 (27.1)	258 (20.5)
“I don’t treat ewes during tupping.”	320 (25.2)	595 (46.9)	174 (13.7)	129 (10.2)	50 (3.9)

Theme 2—Opinions about footrot					
Component 1—Impact of footrot on Productivity (α = 0.698, i:i = 0.325)					
“The number of sheep with this disease in my flock affects how many lambs I produce.”	96 (7.6)	185 (14.7)	297 (23.6)	510 (40.6)	169 (13.4)
“When a sheep has this disease it will lose weight.”	11 (0.9)	11 (0.9)	83 (6.5)	668 (52.4)	503 (39.4)
“When a ewe has this disease she will produce less milk.”	2 (0.2)	29 (2.3)	130 (10.2)	743 (58.5)	367 (28.9)
“The number of sheep with this disease in my flock affects how much money my flock makes.”	22 (1.7)	83 (6.5)	241 (19.0)	640 (50.4)	284 (22.4)
“The number of sheep with this disease in my flock affects how much time I spend on flock management.”	23 (1.8)	62 (4.9)	147 (11.6)	691 (54.4)	348 (27.4)
Component 2—Negative emotions towards footrot (α = 0.773, i:i = 0.531)					
“Having this disease in my flock makes me feel frustrated.”	47 (3.7)	93 (7.4)	272 (21.5)	497 (39.4)	354 (28.0)
“Having this disease in my flock makes me feel miserable.”	85 (6.8)	194 (15.4)	477 (37.9)	360 (28.6)	142 (11.3)
“Having this disease in my flock makes me feel angry.”	166 (13.3)	335 (26.8)	447 (38.1)	192 (15.4)	81 (6.5)
Component 3—Feelings of hopelessness (α = 0.609, i:i = 0.334)					
“There will always be sheep with this disease in my flock.”	99 (7.8)	251 (19.7)	347 (27.2)	478 (37.5)	478 (37.5)
“This disease is very unpredictable.”	38 (3.0)	228 (18.0)	415 (32.7)	467 (36.8)	120 (9.5)
“I am resigned to having lame sheep in my flock.”	189 (15.0)	364 (28.8)	327 (25.9)	324 (25.6)	60 (4.8)
Component 4—Importance of farmer actions/response (α = 0.371, i:i = 0.105)					
“Sheep with this disease will recover on their own in a short time if left untreated.” ^R1^	806 (64.7)	382 (30.7)	27 (2.2)	3 (0.2)	27 (2.2)
“Sheep with this disease will recover in a short time if treated.”	28 (2.3)	97 (7.8)	273 (21.9)	678 (54.4)	170 (13.6)
“Sheep with this disease should be treated within three days of becoming lame.”	17 (1.2)	47 (3.7)	252 (20.0)	657 (52.1)	289 (22.9)
“My actions control how many sheep there are in my flock with this disease.”	21 (1.7)	51 (4.0)	160 (12.7)	704 (55.7)	327 (25.9)
“I have a clear understanding of this disease.”	20 (1.6)	128 (10.1)	444 (35.2)	540 (42.8)	130 (10.3)
Component 5—Traditional methods of treating lameness (α = 0.382, i:i = 0.172)					
“When a sheep is lame with this disease, trimming the foot will delay healing.”	58 (4.6)	115 (9.1)	240 (19.0)	584 (46.1)	269 (21.3)
“Even mildly lame sheep with this disease should be treated with antibiotic injection.”	108 (8.5)	362 (28.6)	263 (20.7)	416 (32.8)	119 (9.4)
“Sheep that are repeatedly lame with this disease should be culled.”	476 (37.5)	592 (46.7)	128 (10.1)	57 (4.5)	15 (1.2)

Theme 3—Knowledge about the causes					
Component 1—Aspects of transmission (α = 0.620, i:i = 0.254)					
“This disease is caused by bacteria.”	6 (0.5)	15 (1.2)	87 (6.8)	781 (61.4)	384 (30.2)
“This disease occurs by chance”. ^R1^	268 (21.3)	535 (42.5)	334 (26.5)	110 (8.7)	12 (1.0)
“This disease is caused by infection in the pasture.”	25 (2.0)	73 (5.8)	258 (20.5)	726 (57.6)	179 (14.2)
“This disease is caused by keeping ewes that are repeatedly lame.”	27 (2.1)	98 (7.7)	290 (22.8)	658 (51.7)	199 (15.6)
“This disease is caused by other sheep with this disease in the flock.”	18 (1.4)	43 (3.4)	178 (14.1)	743 (58.6)	285 (22.5)
Component 2—Pasture and housing (α = 0.612, i:i = 0.243)					
“This disease is caused by long pasture.”	103 (8.2)	481 (38.1)	446 (35.4)	194 (15.4)	37 (2.9)
“This disease is caused by wet pasture.”	32 (2.5)	165 (13.0)	367 (28.9)	603 (47.4)	105 (8.3)
“This disease is caused by high stocking density.”	33 (2.6)	189 (15.0)	455 (36.1)	513 (40.1)	72 (5.7)
“This disease is caused by housing sheep.”	31 (2.5)	212 (16.9)	375 (29.9)	493 (39.4)	142 (11.3)
“This disease is caused by weather conditions.”	37 (2.9)	139 (11.0)	356 (28.1)	647 (51.0)	90 (7.1)
Component 3—Factors that do not cause footrot (α = 0.590, i:i = 0.219)					
“This disease is caused by routine foot trimming of the flock.”	236 (18.7)	628 (49.7)	271 (21.4)	106 (8.4)	23 (1.8)
“This disease is caused by injury to the foot.”	74 (5.9)	341 (27.0)	387 (30.6)	418 (33.1)	44 (3.5)
“This disease is caused by gathering sheep together.”	121 (9.6)	423 (33.6)	423 (33.6)	260 (20.7)	31 (2.5)
“This disease is caused by foot bathing.”	425 (33.5)	646 (50.6)	164 (12.9)	17 (1.3)	17 (1.3)
“This disease is caused by trimming sheep feet until they bleed.”	97 (7.7)	315 (24.9)	429 (33.9)	320 (25.3)	106 (8.4)
Component 4—Factors that do not cause footrot (α = 0.466, i:i = 0.230)					
“This disease is caused by poor body condition.”	261 (20.6)	674 (53.2)	264 (20.9)	55 (4.3)	12 (1.0)
“This disease is caused by soil type on the farm.”	132 (10.4)	410 (32.3)	488 (38.5)	213 (16.8)	26 (2.1)
“This disease is caused by overgrown horn on the feet.”	69 (5.4)	327 (25.8)	352 (27.7)	464 (36.5)	58 (4.6)
Component 5—Genetic susceptibility (α = 0.441, i:i = 0.283)					
“This disease is hereditary, it runs in the family.”	73 (5.8)	250 (19.8)	472 (37.4)	415 (32.9)	52 (4.1)
“This disease is caused by the breed of the sheep.”	144 (11.5)	418 (33.3)	484 (38.6)	179 (14.3)	29 (2.3)
“This disease is caused by a high protein diet.”^a^	136 (10.8)	547 (43.5)	479 (38.1)	83 (6.6)	13 (1.0)

Theme 4					
Empathic Concern (α = 0.661, i:i = 0.22)					
“I often have tender, concerned feelings for people less fortunate than me.”	68 (5.6)	155 (12.7)	354 (29.0)	542 (44.4)	101 (8.3)
“Sometimes I do not feel very sorry for other people when they are having problems.”^b^	422 (34.5)	243 (19.9)	308 (25.2)	199 (16.3)	51 (4.2)
“When I see someone being taken advantage of, I feel kind of protective towards them.”	51 (4.2)	134 (10.9)	240 (19.5)	601 (48.9)	202 (16.5)
“Other people's misfortunes do not usually disturb me a great deal.” b	333 (27.2)	316 (25.8)	358 (29.3)	187 (15.3)	30 (2.5)
“When I see someone being treated unfairly, I sometimes don't feel very much pity for them.” b	617 (50.4)	265 (21.7)	261 (21.3)	64 (5.2)	17 (1.4)
“I am often quite touched by things that I see happen.”	80 (6.5)	190 (15.5)	287 (23.4)	508 (41.5)	160 (13.1)
“I would describe myself as a pretty soft-hearted person.”	86 (7.0)	185 (15.1)	313 (25.5)	480 (39.2)	162 (13.2)
Perspective taking (α = 0.587, i:i = 0.200)					
“I sometimes find it difficult to see things from the other guy's point of view”.^b^	340 (27.6)	292 (23.7)	380 (30.9)	191 (15.5)	27 (2.2)
“I try to look at everybody's side of a disagreement before I make a decision.”	68 (5.5)	159 (12.9)	157 (12.8)	624 (50.7)	222 (18.1)
“I sometimes try to understand my friends better by imagining how things look from their perspective.”	59 (4.8)	179 (14.7)	360 (29.5)	522 (42.7)	102 (8.4)
“If I'm sure I'm right about something, I don't waste much time listening to other people's arguments.” ^R1^	201 (16.4)	268 (21.8)	329 (26.8)	308 (25.1)	121 (9.9)
“I believe that there are two sides to every question and try to look at them both.”	67 (5.5)	134 (10.9)	184 (15.0)	591 (48.1)	253 (20.6)
“When I'm upset at someone, I usually try to put myself in their shoes for a while.”	178 (14.5)	236 (19.2)	441 (35.9)	325 (26.4)	49 (4.0)

^R1^Item reverse scored prior to inclusion of PCA.

a Did not improve the internal consistency of any component and so was kept as an isolated statement.

b Item reverse scored before calculating subscale.

**Table 2:** Number and percentage of 1294 English sheep farmers by management of lameness/footrot and the geometric mean (95% CI) percentage lameness in ewes.

Variable	Number of farmers	Percentage of farmers	GM (%) Lameness in ewes (95% CI)
Catch and treat sheep within 3 days of seeing lame			
No	632	49.6	4.3 (4.1–4.6)
Yes	641	50.4	3.2 (3.0–3.4)

Number of sheep lame in a group before catching and treating			
1	180	14.3	2.5 (2.1–2.8)
2–5	657	52.2	3.5 (3.3–3.7)
6+	422	33.5	4.8 (4.5–5.3)

Never foot trim feet of lambs with footrot^a^			
No	922	84.1	4.0 (3.8–4.2)
Yes	174	15.9	3.0 (2.6–3.4)

Correctly diagnosed interdigital dermatitis and footrot			
No	242	19.5	3.9 (3.5–4.4)
Yes	1002	80.5	3.7 (3.4–3.9)

Always treat ewes with footrot with antibiotic injection			
No	906	75.6	3.9 (3.7–4.1)
Yes	293	24.4	3.3 (3.0–3.7)

Always spray feet of ewes with footrot			
No	423	34.7	3.7 (3.4–4.0)
Yes	795	65.3	3.8 (3.6–4.0)

Remember lame sheep for culling by memory			
No	1120	86.6	3.6 (3.4–3.8)
Yes	174	13.4	4.3 (3.9–4.9)

Number of episodes of lameness before culling			
Did not cull	671	54.6	3.6 (3.3–3.8)
One	40	3.3	1.8 (1.2–2.8)
Two	151	12.3	3.4 (3.0–3.9)
>Two	323	26.3	4.3 (4.0–4.7)
Persistently lame	45	3.7	4.5 (3.6–5.6)

Vaccinate ewes against footrot			
No	1080	83.5	3.8 (3.6–4.0)
Yes	214	16.5	3.2 (2.8–3.6)

a “never trim feet of lambs” was used as a proxy measure for foot trimming behaviour in general. Given that only 4% of farmers reported to “never trimming feet of ewes”, this proxy measure was considered more robust for inclusion into the LCA.

**Table 3:** Multivariable multinomial regression model for attitudes, emotions and personalities by latent class membership for 1294 English sheep farmers.

Theme and component (Supplementary Table 1)	Latent class	RRR	95% CI
Theme 1: Barriers to treating footrot			
Component 1: Production cycle barriers to treatment	LC2	1.36	1.04–1.78
	LC3	1.25	0.94–1.65

Theme 2: Opinions about footrot			
Component 2: Negative emotions towards footrot	LC2	1.17	0.93–1.48
	LC3	1.39	1.09–1.77
Component 5: Traditional methods of treating lameness	LC2	2.94	2.17–4.00
	LC3	2.17	1.59–3.03

Theme 3: Knowledge about the causes			
Component 1: Aspects of transmission	LC2	0.64	0.41–0.99
	LC3	1.07	0.68–1.69

**Table 4:** Multivariable negative binomial regression model for the relationship between attitudes, emotions and personalities and period prevalence of lameness in sheep May 2012–April 2013 for 1294 English sheep farmers.

Theme and component/personality trait	IRR	95% CI
Theme 1: Barriers to treating footrot		
Component 2: Production cycle barriers to treating footrot	1.13	1.06–1.20

Theme 2: Opinions about footrot		
Component 2: Negative emotions towards footrot	1.13	1.08–1.20
Component 3: Feelings of hopelessness towards footrot	1.20	1.13–1.28
Component 4: Importance of farmer actions/response	0.76	0.68–0.84

Theme 5: Farmer personality		
Conscientiousness	0.95	0.90–1.00
