# Supplementary material for: Associations between sheep farmer attitudes, beliefs, emotions and personality, and their barriers to uptake of best practice: The example of footrot
Source: Prev Vet Med. 2017 Apr 1;139(Pt B):123–33. doi: 10.1016/j.prevetmed.2016.05.009 (PMC5409802; doi:10.1016/j.prevetmed.2016.05.009)
Supplement: Supplementary file 1 [file mmc1.docx]

**Supplementary Figure 1**

Distribution of personality scores for approx. 1294 English sheep farmers in 2013

**Supplementary Table 1**

Univariable multinomial regression model for attitudes, emotions and personalities by latent class membership for 1294 English sheep farmers

| **Theme and component/personality trait**  **(Table 4)** | **Latent Class** | **RRR** | **95% CI** |
| --- | --- | --- | --- |
| **Theme 1: Barriers to treating footrot** |  |  |  |
| Component 1: Practical barriers to treatment | LC2 | 1.14 | 0.91 – 1.43 |
|  | LC3 | **1.26** | 0.99 – 1.60 |
| Component 2: Production cycle barriers to treatment | LC2 | **1.49** | 1.16 – 1.91 |
|  | LC3 | **1.26** | 0.97 – 1.64 |
| **Theme 2: Opinions about footrot** |  |  |  |
| Component 1: Impact of footrot on Productivity | LC2 | **0.70** | 0.51 – 0.97 |
|  | LC3 | 1.18 | 0.84 – 1.67 |
| Component 2: Negative emotions towards footrot | LC2 | **0.86** | 0.70 – 1.07 |
|  | LC3 | 1.15 | 0.92 – 1.44 |
| Component 3 : Feelings of hopelessness | LC2 | **1.32** | 1.05 – 1.67 |
|  | LC3 | **1.40** | 1.10 – 1.79 |
| Component 4: Importance of farmer actions/ response | LC2 | **0.63** | 0.40 – 0.97 |
|  | LC3 | **0.55** | 0.35 – 0.87 |
| Component 5 : **Traditional methods of treating lameness** | LC2 | **3.33** | 2.5 – 4.35 |
|  | LC3 | **2.08** | 1.56 – 2.78 |
| **Theme 3: Knowledge about the causes** |  |  |  |
| Component 1: Aspects of transmission | LC2 | **0.53** | 0.36 – 0.77 |
|  | LC3 | 1.00 | 0.67 – 1.48 |
| Component 2: Pasture and housing | LC2 | 1.17 | 0.85 – 1.61 |
|  | LC3 | **1.35** | 0.96 – 1.90 |
| Component 3: Factors that do not cause footrot | LC2 | **0.65** | 0.47 – 0.90 |
|  | LC3 | 0.89 | 0.63 – 1.25 |
| Component 4: Factors that do not cause footrot | LC2 | **1.28** | 0.96 – 1.70 |
|  | LC3 | **1.24** | 0.91 – 1.67 |
| Component 5 : Genetic susceptibility | LC2 | **0.80** | 0.62 – 1.02 |
|  | LC3 | 1.06 | 0.82 – 1.37 |
| “This disease is caused by a high protein diet.” | LC2 | 1.00 | 0.80 – 1.27 |
|  | LC3 | 1.16 | 0.91 – 1.48 |
| **Theme 4: Empathy** |  |  |  |
| Empathic Concern | LC2 | 1.00 | 0.96 – 1.05 |
|  | LC3 | 0.96 | 0.92 – 1.01 |
| Perspective Taking | LC2 | 0.99 | 0.94 – 1.05 |
|  | LC3 | 0.99 | 0.94 – 1.05 |
| **Theme 5: Farmer personality** |  |  |  |
| Extraversion | LC2 | 1.05 | 0.87 – 1.27 |
|  | LC3 | 1.03 | 0.84 – 1.26 |
| Agreeableness | LC2 | 1.01 | 0.83 – 1.23 |
|  | LC3 | 0.96 | 0.78 – 1.18 |
| Conscientiousness | LC2 | 1.02 | 0.81 – 1.27 |
|  | LC3 | 1.03 | 0.81 – 1.30 |
| Emotional Stability | LC2 | 1.05 | 0.86 – 1.28 |
|  | LC3 | 0.96 | 0.78 – 1.19 |
| Openness to experiences | LC3 | 0.91 | 0.73 – 1.13 |
|  | LC2 | 1.00 | 0.80 – 1.25 |

**Supplementary Table 2**

Univariable negative binomial regression model for the relationship between attitudes, emotions and personalities and period prevalence of lameness in sheep between May 2012 - April 2013 for 1294 English sheep farmers.

| **Theme and component/personality trait**  **(Table 4)** | **IRR** | **95% CI** |
| --- | --- | --- |
| **Theme 1: Barriers to treating footrot** |  |  |
| Component 1: Practical barriers to treatment | **1.11** | 1.05 – 1.17 |
| Component 2: Production cycle barriers to treatment | **1.15** | 1.08 – 1.22 |
| **Theme 2: Opinions about footrot** |  |  |
| Component 1: Impact of footrot on Productivity | 1.02 | 094 – 1.10 |
| Component 2: Negative emotions towards footrot | **1.14** | 1.09 – 1.20 |
| Component 3: Feelings of hopelessness | **1.24** | 1.17 – 1.31 |
| Component 4: Importance of farmer actions/ response | **0.74** | 0.67 – 0.82 |
| Component 5: Traditional methods of treating lameness | 1.00 | 0.94 – 1.08 |
| **Theme 3: Knowledge about the causes** |  |  |
| Component 1: Aspects of transmission | 0.96 | 0.88 – 1.04 |
| Component 2: Pasture and housing | **1.16** | 1.07 – 1.25 |
| Component 3: Factors that do not cause footrot | 1.00 | 0.92 – 1.07 |
| Component 4: Factors that do not cause footrot | **1.07** | 1.00 – 1.14 |
| Component 5: Genetic susceptibility | 1.01 | 0.95 – 1.08 |
| **Theme 4: Empathy** |  |  |
| Empathic Concern | 0.99 | 0.99 – 1.01 |
| Perspective taking | 0.99 | 0.98 – 1.00 |
| **Theme 5: Farmer personality** |  |  |
| Extraversion | 1.02 | 0.97 – 1.06 |
| Agreeableness | 1.00 | 0.95 – 1.05 |
| Conscientiousness | 0.96 | 0.91 – 1.01 |
| Emotional Stability | 0.99 | 0.94 – 1.03 |
| Openness to experiences | 0.97 | 0.93 – 1.02 |

**Supplementary Table 3**

Goodness of fit statistics for each latent class model

| Number of classes | Fit statistics | | | | | |
| --- | --- | --- | --- | --- | --- | --- |
|  | AIC | BIC | Entropy | H_1_ vs H_0_ | LMR | BLRT |
| 2 | 14282.564 | 14422.032 | 0.746 | 3 class vs 2 class | 0.05 | <0.001 |
| 3 | 14215.624 | 14427.410 | 0.697 |  |  |  |
| 4 | 14174.006 | 14458.109 | 0.591 | 4 class vs 3 class | 0.40 | N/A |

*AIC, Akaike’s Information Criteria; BIC, Bayes’ Information Criteria; LMR, Lo-Mendell-Rubin test; BLRT, bootstrapped likelihood ratio test.

**Supplementary Table 4**

Distribution of 1294 English sheep farmers by latent class and conditional probabilities* (standard error) for each behaviour by latent classes

|  | | Latent Class PROBABILITY (SE) | | |
| --- | --- | --- | --- | --- |
|  |  | LC1  Best Practice  138 (11%) | LC2  Slow to act  738 (57%) | LC3  Slow to act, delayed culling  418 (32%) |
| Geometric mean prevalence of lameness (%) | | 2.95 | 3.60 | 4.10 |
| **Variable** | |  |  |  |
| Catch within 3 days of seeing lame | | **0.900**  (0.110) | **0.491**  (0.037) | **0.320**  (0.058) |
| Number lame to catch and treat | 1 | **0.412**  (0.127) | **0.130**  (0.023) | 0.025  (0.021) |
|  | 2-5 | **0.470**  (0.077) | **0.540**  (0.022) | **0.517**  (0.032) |
|  | 6+ | 0.119  (0.103) | **0.331**  (0.027) | **0.457**  (0.039) |
| Never trim lambs with footrot | | **0.385**  (0.120) | **0.133**  (0.020) | **0.092**  (0.021) |
| Correctly diagnosed ID and footrot | | **0.838**  (0.049) | **0.768**  (0.021) | **0.855**  (0.022) |
| Always injects ewes with footrot | | **0.504**  (0.108) | **0.161**  (0.032) | **0.259**  (0.050) |
| Always spray feet of ewes with footrot | | **0.702**  (0.101) | **0.634**  (0.027) | **0.661**  (0.040) |
| Mark sheep for culling by memory | | **0.153**  (0.055) | **0.040**  (0.009) | **0.296**  (0.038) |
| Episodes of lameness before culling | |  |  |  |
|  | Didn’t cull | **0.308**  (0.147) | **0.909**  (0.040) | 0.000  (0.000) |
|  | Once | **0.099**  (0.047) | **0.029**  (0.011) | 0.004  (0.000) |
|  | Twice | **0.188**  (0.062) | 0.062  (0.039) | **0.201**  (0.056) |
|  | >Twice | **0.379**  (0.102) | 0.000  (0.000) | **0.686**  (0.040) |
|  | Persistent | 0.026  (0.026) | 0.000  (0.000) | **0. 110**  (0.022) |
| Vaccinate ewes against footrot | | **0.220**  (0.058) | **0.133**  (0.015) | **0.196**  (0.026) |

*Probability that a farmer allocated to the latent class will perform that behaviour
